# Simplified Paper Format for Detecting HIV Drug Resistance in Clinical Specimens by Oligonucleotide Ligation

**DOI:** 10.1371/journal.pone.0145962

**Published:** 2016-01-11

**Authors:** Nuttada Panpradist, Ingrid A. Beck, Michael H. Chung, James N. Kiarie, Lisa M. Frenkel, Barry R. Lutz

**Affiliations:** 1 Department of Bioengineering, University of Washington, Seattle, Washington, United States of America; 2 Seattle Children’s Research Institute, Seattle, Washington, United States of America; 3 Department of Internal Medicine, University of Washington, Seattle, Washington, United States of America; 4 Department of Global Health, University of Washington, Seattle, Washington, United States of America; 5 Departments of Obstetrics and Gynecology, University of Nairobi, Nairobi, Kenya; 6 Departments of Pediatrics and Laboratory Medicine, University of Washington, Seattle, Washington, United States of America; New York University, UNITED STATES

## Abstract

Human immunodeficiency virus (HIV) is a chronic infection that can be managed by antiretroviral treatment (ART). However, periods of suboptimal viral suppression during lifelong ART can select for HIV drug resistant (DR) variants. Transmission of drug resistant virus can lessen or abrogate ART efficacy. Therefore, testing of individuals for drug resistance prior to initiation of treatment is recommended to ensure effective ART. Sensitive and inexpensive HIV genotyping methods are needed in low-resource settings where most HIV infections occur. The oligonucleotide ligation assay (OLA) is a sensitive point mutation assay for detection of drug resistance mutations in HIV *pol*. The current OLA involves four main steps from sample to analysis: (1) lysis and/or nucleic acid extraction, (2) amplification of HIV RNA or DNA, (3) ligation of oligonucleotide probes designed to detect single nucleotide mutations that confer HIV drug resistance, and (4) analysis via oligonucleotide surface capture, denaturation, and detection (CDD). The relative complexity of these steps has limited its adoption in resource-limited laboratories. Here we describe a simplification of the 2.5-hour plate-format CDD to a 45-minute paper-format CDD that eliminates the need for a plate reader. Analysis of mutations at four HIV-1 DR codons (K103N, Y181C, M184V, and G190A) in 26 blood specimens showed a strong correlation of the ratios of mutant signal to total signal between the paper CDD and the plate CDD. The assay described makes the OLA easier to perform in low resource laboratories.

## Introduction

Since human immunodeficiency virus (HIV) was recognized in the 1980s [1], 75 million people have become infected, of which about half have died of AIDS [2]. In 1996 combination antiretroviral treatment (ART) was found to control HIV replication and to prolong lives of infected individuals [3]. However, lifelong ART is required, and if intracellular drug concentrations fall to subtherapeutic levels, HIV drug-resistant (DR) strains are selected. Transmission of DR virus can undermine ART efficacy. Therefore, testing of individuals prior to initiation of treatment is recommended in North America and Europe. HIV infections are most prevalent in low- to middle-income countries where transmission of resistant viruses have increased over the past decade [4–9]. In the absence of proper DR screening, the spread of DR HIV can reduce the durability of available first-line ART [10,11] and as a result there is an urgent need to establish DR HIV testing prior to initiation of ART internationally [12]. In addition, DR testing in patients failing first-line therapy may assist in treatment management in low-resource settings, where available drugs are limited and unnecessary switches to second-line should be avoided. However clinical trials are needed to assess the value of this approach.

In North America and Europe, consensus sequencing (Sanger) has become the standard method to screen for HIV drug resistance. A genotype of the HIV *pol* encoding protease and reverse transcriptase currently costs US$200-$700, which is unaffordable to most patients in low- to middle-income countries with the highest prevalence of HIV. In addition, population-based sequencing only detects DR when the proportion of mutant virus is >15%-30% of the total viral population [13], while lower proportions can result in treatment failure [14]. Whereas ultra-deep sequencing can detect DR as low as 0.5–1% of the viral population, this technology is more complex and uses more expensive instruments compared to Sanger sequencing [15].

Consensus sequencing provides the genotype over the entire HIV *pol* region amplified, which may include protease, reverse transcriptase and integrase, and therefore simultaneously assesses all HIV drug resistance sites within the amplified region. However, in settings where the availability of antiretrovirals is limited, drug resistance screening can be accomplished by targeting a small set of specific single nucleotide mutations associated with resistance to antiretrovirals used in the community.

**Fig 1:**
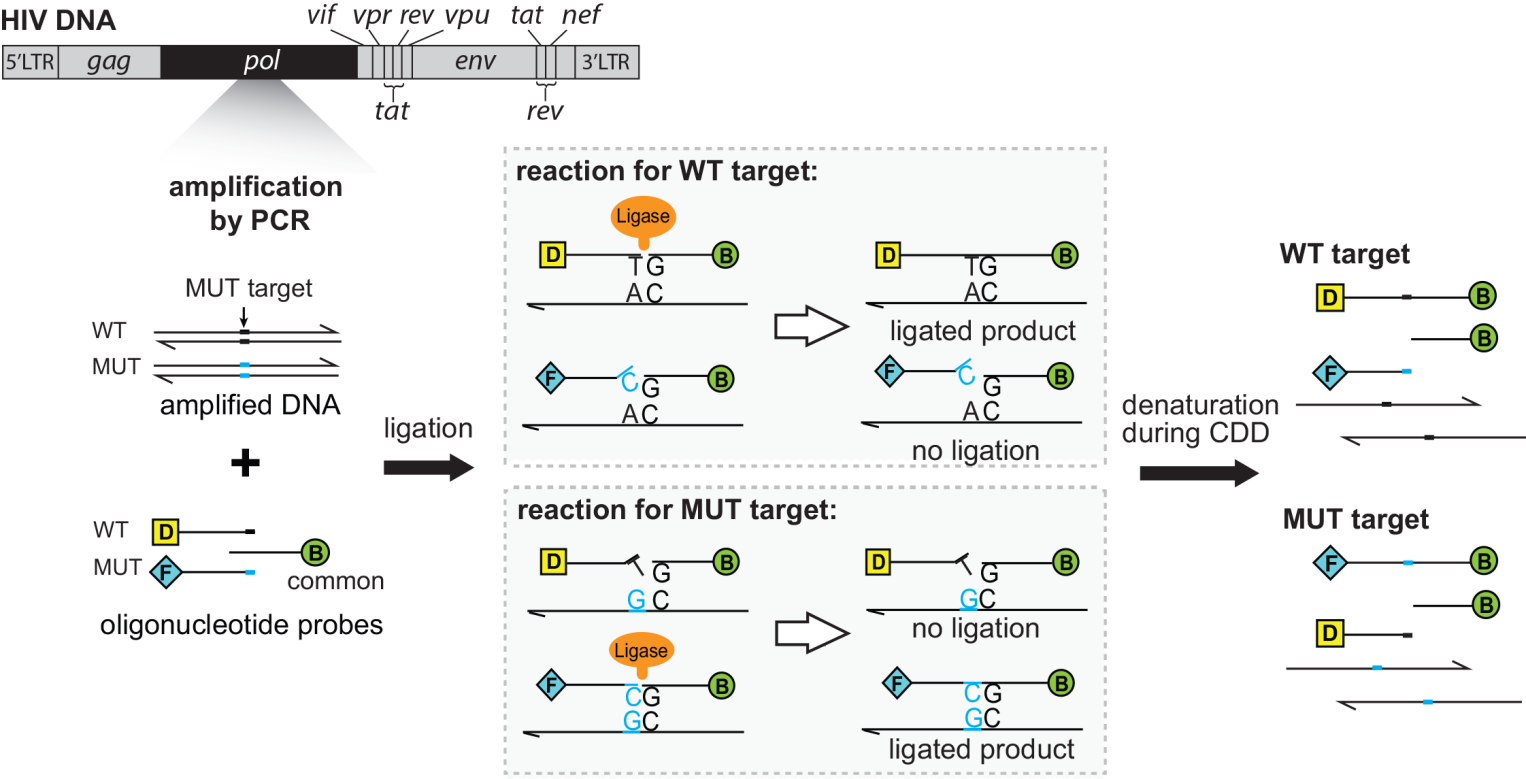
Schematic of amplification and ligation in the existing oligonucleotide ligation assay (OLA). The *pol* gene of HIV DNA is amplified by nested PCR. Note that the HIV DNA gene map positions are not to scale. A portion of the amplicon is mixed with three oligonucleotide probes: a 5’ fluorescein (F) -conjugated mutant (MUT)-specific HIV probe; a 5’ digoxigenin (D) -conjugated wild-type (WT)-specific probe; and a 5’ phosphorylated, 3’ biotin (B) -conjugated common probe. When specific probes are complementary at the mutation site, they are ligated to the common probe to create a DNA strand with labels at both ends. Only ligated products are detected during the CDD procedure (surface **c**apture, **d**enaturation of oligonucleotide from target DNA, and enzyme-based detection).

In 1988, the oligonucleotide ligation assay (OLA) was first developed to diagnose mutations associated with sickle-cell anemia [16] and was then adapted to detect drug resistance mutations in HIV [17–21]. The OLA has been developed and validated for detection of multiple HIV mutations associated with resistance to protease and reverse transcriptase inhibitors [17–19], it is highly sensitive for detection of minority mutant variants (2–5% of an individual’s HIV population) [21,22], it is tolerant of genetic polymorphisms in clinical specimens [17], and can be adapted to regional variants [23]. The OLA has been used to study HIV drug resistance in various clinical scenarios in Thailand and Africa [6,24] and currently is being tested in a clinical trial (clinicaltrials.gov #NCT01898754). OLA takes advantage of probe hybridization and absolute specificity of ligation to identify a specific base mutation (Fig 1). Two DNA probes hybridize adjacent to one another, with the 3’-end of the upstream probe annealing to the site of the mutation of interest. The probes are then covalently linked by a thermostable ligase only when there is perfect complementation with the template at the mutation site and adjacent 1–3 bases. Performing the ligation at relatively low temperature (37°C) minimizes the effects of polymorphisms within the target sequence, while maintaining the specificity at the site of interest.

**Fig 2:**
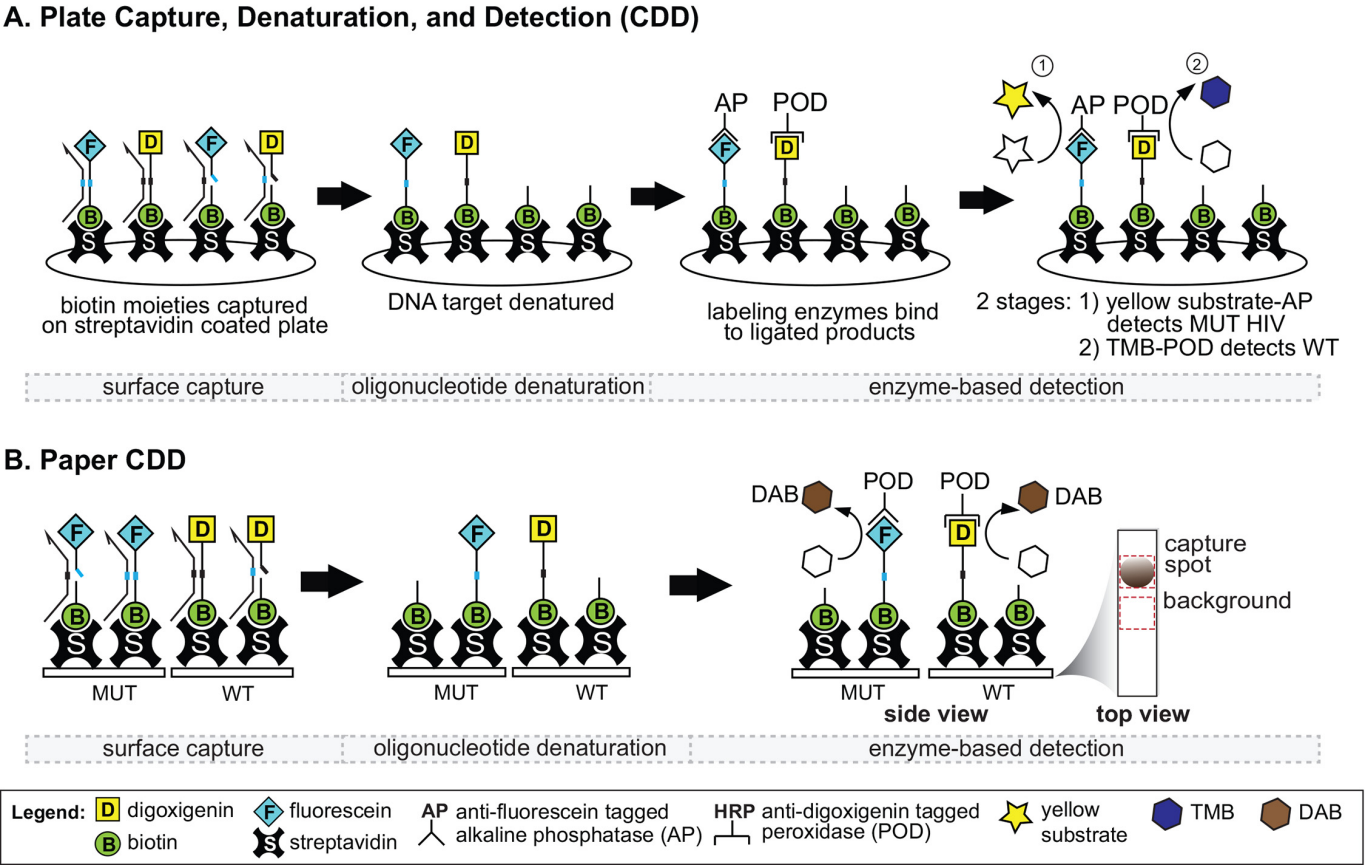
Plate and paper formats for Capture, Denaturation and Detection (CDD) in the oligonucleotide ligation assay. (A) Plate CDD procedure. Products from the ligation step (both ligated and non-ligated products) are captured on a streptavidin-coated plate. Non-ligated probes are released during oligonucleotide denaturation, and ligated MUT and WT probes are then detected in sequential enzyme-based immunoassays (labeling by different detection antibodies, alkaline phosphatase yellow substrate development, optical density reading at 405nm, wash steps, tetramethylbenzidine (TMB) development, stop solution, and optical density reading at 450nm). (B) Paper CDD procedure. Similar to the plate CDD procedure, products from the ligation step (both ligated and non-ligated products) are captured. However, here the products are captured on paper strips by immobilized streptavidin. Non-ligated probes are released during oligonucleotide denaturation. Antibodies targeting the end-labels of the mutant (MUT) or wild-type (WT) probes have conjugated horseradish peroxidase (POD) that converts 3,3’ diazoaminobenzidine substrate (DAB) into brown precipitates. Signals were captured by the scanner (600 DPI). Reported signals represent capture spot intensity minus a background region from the strip.

Following the ligation step, the ligated probes are detected via surface capture, denaturation of oligonucleotide from target DNA, and enzyme-based detection (CDD, Fig 2A). Ligation and CDD are performed after PCR amplification and can be used to target either HIV DNA or viral RNA. Probe sets specific for the wild-type (WT) and the mutant (MUT) genotypes provide estimates of their relative proportion that is comparable to quantification by 454-pyrosequencing [22].

The OLA-based DR HIV test offers an attractive cost (<$5.00 per codon) and a higher sensitivity compared to Sanger sequencing. However, the duration and complexity of the procedure, in particular the CDD procedure, has made it difficult to transfer OLA to resource-limited laboratories [17]. The goal of this work was to simplify the CDD procedure by adapting it to a paper strip format (Fig 2B). The CDD chemistry was directly transferred onto the paper format with modifications that reduced the number of steps and total assay time, and allowed visual detection of results. The paper-format CDD was used to identify mutants at four common HIV DR codons in a blinded set of blood specimens, and the results were compared to results obtained with the current plate CDD.

## Materials and Methods

### Study design and specimens

Leftover blood specimens from HIV-infected Kenyans collected as part of an observational study of transmitted drug resistance conducted in Kenya in 2010 (unpublished) were tested and analyzed for a retrospective study approved by Human Subjects Protection Committees at Seattle Children’s Hospital and Kenyatta National Hospital. Specimens collected over a period of 2–12 month from subjects with virologic failure of first-line ART were selected based on previous testing by the current plate-format OLA. The selected specimens included HIV subtypes A (N = 8), C (N = 1), D (N = 5), AE (N = 9) and G (N = 3), and one or more drug resistance mutations at four codons in HIV reverse transcriptase (K103N, Y181C, G190A and M184V) spanning a wide range of frequencies in each subjects’ HIV population. The paper CDD was compared to plate CDD by testing the same set of PCR amplicons from participant specimens as well as plasmid standards containing the mutations of interest. The probe hybridization and ligation step was performed separately and at different times for the plate and paper CDD using the same protocol and reagents. Laboratory personnel were “blinded” to the mutational content of each specimen during testing of the paper CDD.

### DNA extraction

Participant’s PBMCs were isolated from 10mL whole blood by gradient centrifugation in Histopaque (Sigma, 1077, St. Louis, MO). Genomic DNA was then extracted from frozen PBMC pellets (~ 5x10^6^ cells) using a DNA Archive Pure DNA Purification System (5 PRIME, Inc., Gaithersburg, MD).

### PCR amplification of HIV pol

#### Clinical specimens

A 1009 bp region of the *pol* gene was amplified by nested PCR. Two μg of each DNA were added to a 50μl reaction mixture containing 400nM of forward (5’-CCT ACA CCT GTC AAC ATA ATT GG-3’) and reverse primers (5’-AAC TTC TGT ATA TCA TTG ACA GTC CA-3’), 200μM dNTPs, 3mM MgCl_2_, and 2.5U of MyTaq^TM^ DNA polymerase (Bioline USA, Inc., Taunton, MA). Cycling conditions included a denaturation cycle of 5 minutes at 94°C and 35 cycles of 30 seconds at 94°C, 30 seconds at 55°C, and 1 minute at 72°C, followed by an extension step of 7 minutes at 72°C. Nested PCR was performed by adding 2μl of first round amplicon to a 50μl reaction mixture containing 400nM of inner forward primer (5’-CAA ATC ACT CTT TGG CAR CGA CC-3’) and inner reverse primer (5’-CAY TTG TCA GGA TGG AGT TCA TA-3’), 200μM dNTPs, 3mM MgCl_2_, and 2.5U of MyTaq DNA polymerase. Second round cycling conditions were the same as the first round, except the extension time was reduced to 30 seconds. Second-round PCR products were visualized by electrophoresis on 1% agarose gels (LE Quick Dissolve Agarose, GeneMate, Kaysville, UT) in SB buffer (5mM sodium tetraborate decahydrate).

#### Plasmid standards

Plasmid DNA containing patient-derived HIV *pol* sequences with mutations at codons of interest served as standards. Plasmid standards for each of the four codons tested were prepared by mixing mutant (MUT) plasmid with wild-type (WT) plasmid (concentrations of 0%, 5%, and 50% MUT). Plasmid standards (0.1ng) were amplified in a 50μl reaction mixture containing forward and reverse M13 primers using the same reagents and conditions as the second round PCR used for clinical specimens. PCR products were visualized by agarose gel electrophoresis.

### Ligation

Sequences and concentrations of ligation probes (S1 Table) for each mutation tested were optimized for HIV subtypes prevalent in Kenya (A, D and C). Ligation reactions were carried out in separate wells for each codon to avoid competition between overlapping probes. DNA amplicons were diluted 1:4 in nuclease-free water (Promega, P1193, Madison, WI). Two μl of diluted DNA were added to 20μl reaction mixture containing: 0.67U of thermostable Ampligase ligase (Epicentre Technology, A3210K, Madison, WI), 12.5mM KCl, 1mM NAD (Sigma, 7004, St. Louis, MO), 1X ligase buffer (20mM Tris pH 8.0, 10mM MgCl_2_, 1mM DDT), 0.1075% Triton-X 100, and the oligonucleotide probes specific for each mutation tested (Integrated DNA Technology, Coralville, IA). The mixture was subjected to 10 cycles of 94°C for 30 seconds, followed by 37°C for 4 minutes, followed by inactivation of the ligase enzyme by addition of 10μl of 100mM EDTA/0.1% Triton X-100.

### Oligonucleotide Capture, Denaturation, and Detection (CDD)

#### Plate-format CDD

The product from the ligation step (32μL) was transferred to a streptavidin-coated 96-well microtiter plate (Sigma, 1173477600, St. Louis, MO)) and covered with an adhesive seal and incubated for one hour at room temperature without being shaken. The plate was then washed twice with 1X denaturing buffer (0.01N sodium hydroxide, 0.05% Tween-20) followed by two washes with 1X neutralization buffer (100mM Tris pH 7.5, 150mM sodium chloride, 0.05% Tween-20). Fifty μl of an antibody mixture (37.5mU anti- fluorescein-alkaline phosphatase (AP) Fab fragments (Roche, 11426338910, Indianapolis, IN) and 7.5mU anti-digoxigenin-peroxidase (POD) Fab fragments (Roche, 11207733910, Indianapolis, IN) in 1X Phosphate Buffered Saline (1X PBS) with 0.5% Bovine Serum Albumin (Sigma, A6793, St. Louis, MO) was added to each well and incubated for 30 minutes at room temperature in the dark. The plate was then washed six times with 1X neutralization buffer. Subsequently, 100μl of alkaline phosphate yellow liquid substrate (Sigma, P7998, St. Louis, MO) were added and incubated until the yellow color developed fully (20 to 35 minutes, depending on the mutation tested), and the optical density (OD) was read at 405nm (SpectraMax 190, Molecular Devices, Sunnyvale, CA) to measure the MUT signal level. The plate was then washed six times with 300μl of 1X neutralization buffer, and 50μl of tetramethylbenzidine (TMB) One Solution (Promega, G7431, Madison, WI) were added to each well. After 3–5 minutes at room temperature, 50μl of 0.3M H_2_SO_4_ were added to each well to stop the reaction. The OD was read at 450nm to measure the WT signal level.

The WT OD or MUT OD (range: 0–4 A.U.) for each specimen was calculated from the OD of the sample wells minus the average OD of the blank wells (enzyme substrates only). The WT OD and MUT OD from a sample was then converted to a single value as MUT Ratio (MUT OD/(MUT OD + WT OD)), in order to account for rare cases of differential probe-binding efficiency between plasmid standards and clinical specimens [22]. The MUT Ratios of all plate CDD experiments are summarized in S2 Table.

#### Paper-format CDD

A Mylar-backed nitrocellulose membrane (EMD Millipore, High-Flow Plus 13504XSS, Billerica, MA) was cut into 3mm x 25mm strips using a CO_2_ laser cutter (Universal Laser Systems, Scottsdale, AZ) at 5% speed, 5% power, and 1000 pulses per inch. The strips were hand-spotted with 1μl of 1mg/ml streptavidin (Sigma, S-0677, St. Louis, MO) in 1X PBS (Corning, 21-040-CV, Manassas, VA), incubated 2 hours at 37°C (Thermo Forma Water Jacketed CO_2_ incubator), and kept dried in a desiccator (Bel-Art 42073100, Wayne, NJ) at 20% humidity, 22–25°C. A cellulose absorbent pad (EMD Millipore, CF22300, Billerica, MA) was attached to the nitrocellulose strips with a 3-mm overlap via an adhesive plastic backing (10 mil Melinex with adhesive on 1 side, Fraylock, San Carlos, CA).

Paper devices were used within 3 days of reagent spotting. The strips were dipped in a timed sequence (5 minutes each) in a series of untreated polystyrene 96-well microtiter plate (Sigma, P7491-1CS, St. Louis, MO) containing the following reagents: (1) 25μl 1% BSA/1X PBS buffer (Bovine Serum Albumin–Sigma, A3059-1006, St. Louis, MO), (2) 27.5μl ligated products, (3) 25μl 10X denaturing solution (0.1N sodium hydroxide with 0.05% Tween-20), (4) 25μl 1X neutralizing buffer, (5) 25μl of labeling antibodies (3.75U of anti-digoxigenin-POD (Roche, 11207733910, Indianapolis, IN) for WT detection or anti-fluorescein-POD (Roche, 11426346910, Indianapolis, IN) for MUT detection), (6) 25μl of 1X PBS with 0.05% Tween-20 (PBST), (7) 40μl of signal amplification reagent (250μg/ml 3,3’-diaminobenzidine (DAB), KPL, 710008, Gaithersburg, MD) with 250μg/ml w/v sodium percarbonate (Sigma, 371432, St. Louis, MO) in 1X PBST, and (8) 25μl of 1X PBST. Wet strips were immediately imaged (48 BitHDR, 600dpi, non-gamma corrected, no filter) using a flatbed scanner (Epson V700, Long Beach, CA) and SilverFast software (LaserSoft Imaging, Sarasota, FL). The humidity and temperature during the experiments were not controlled but were measured (45–50% humidity and 22–25°C). The WT and MUT signals were extracted from scanned images of the paper strips using in-house codes written in MATLAB (MathWorks, Natick, MA). Intensity of each pixel from the capture spot (specimen) and a region upstream of the capture spot (background) were extracted from two regions of interest (ROIs) of equal size (55 x 40 pixels, 2.32mm x 1.70mm). The reported signal intensity was calculated as the 95^th^ percentile of the intensity in the capture spot ROI minus the 95^th^ percentile of the background ROI. S3 Table provides the detailed calculation and the MATLAB code. The MUT Ratio from a sample evaluated by paper CDD was calculated from MUT signal intensity/(MUT signal intensity + WT signal intensity). The MUT Ratios of all paper CDD experiments are summarized in S4 Table.

## Results

**Fig 3:**
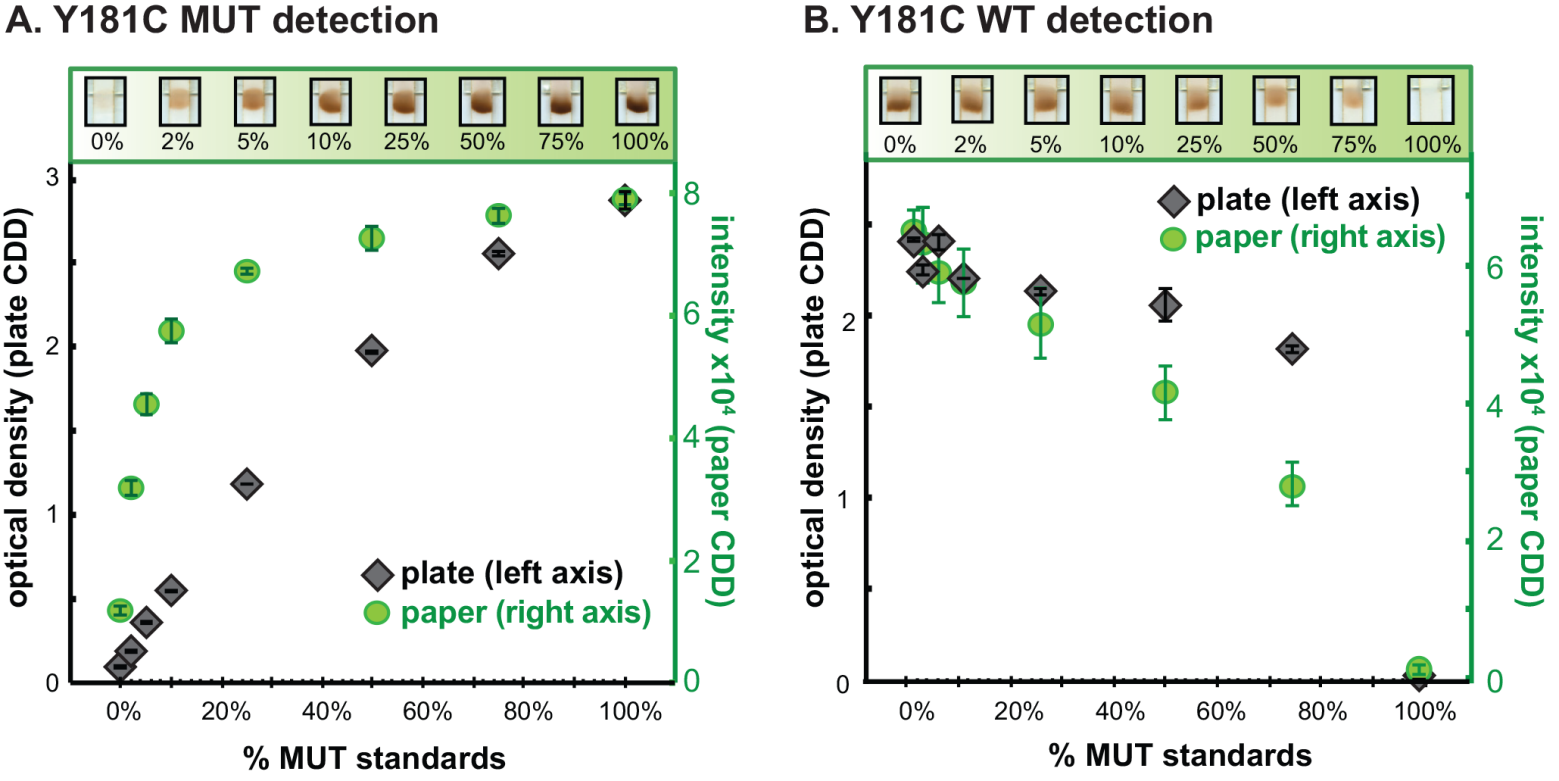
Comparison of paper Capture, Denaturation, and Detection (CDD) and plate CDD for analysis of a plasmid standard mixture series for mutation Y181C. (A) Scanned images of paper CDD MUT (left) and WT (right) detection (B) Mutant (MUT) detection (C) wild-type (WT) detection. Left axes: Specimen optical density (OD) minus negative control OD analyzed in duplicate by plate CDD (mean ± SE). Right axes: Specimen capture intensity minus background intensity analyzed in triplicate by paper CDD (mean ± SE). The ratio of mean signal intensities for 2% MUT and 0% MUT was 2.00 for plate CDD and 2.74 for paper CDD.

In the current OLA using the plate-format CDD, conditions are optimized to obtain a sensitive MUT response to samples with low % MUT, while the WT assay gives saturated signals in the range of 0–50% MUT to serve as a positive control for successful ligation and detection. To investigate the analytical response of OLA using the paper CDD, a plasmid mixture dilution series for the Y181C mutation (0, 2, 5, 10, 25, 50, 75, 100% MUT) was tested for MUT and WT responses and compared to the signals obtained with the plate CDD (Fig 3). The paper CDD showed strong MUT signal for 2% MUT, but its signal saturated at a lower % MUT than in the plate CDD (Fig 3A and 3B). The paper CDD showed strong WT signals across the full dilution series, as in the plate CDD (Fig 3B). Correlation plots of MUT data in Fig 3A, WT data in Fig 3B, and MUT Ratios from plate CDD and from paper CDD are shown in S1 Fig.

**Fig 4:**
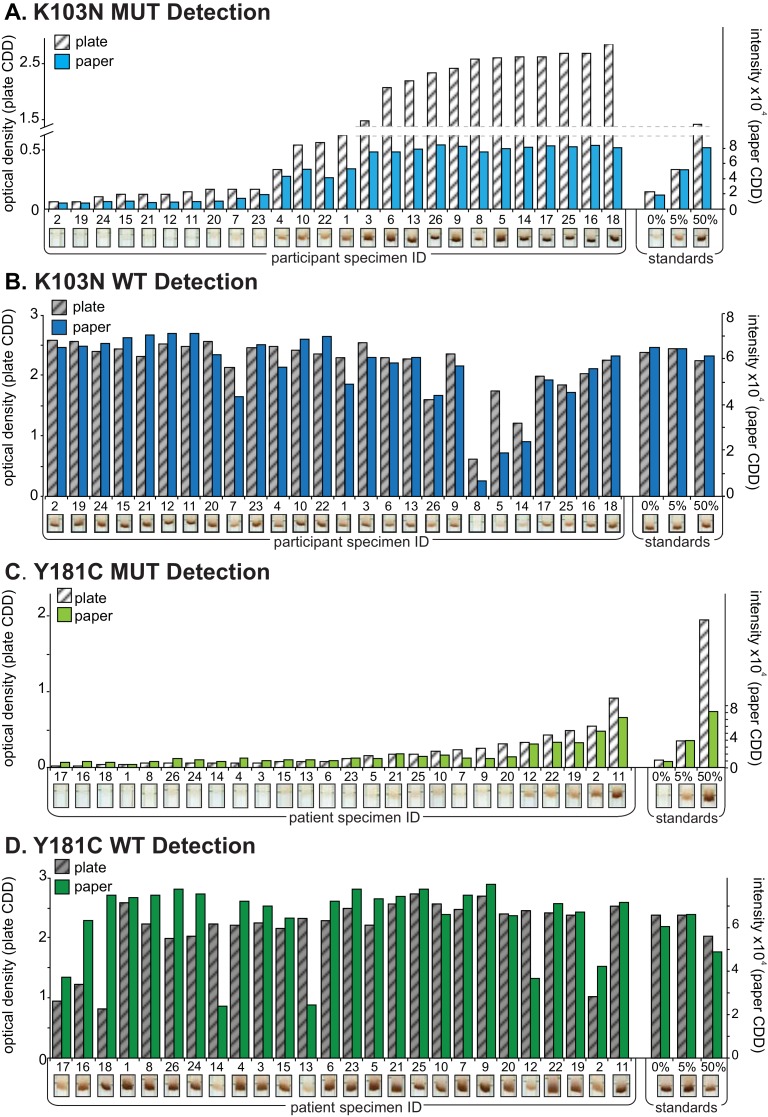
Analysis of clinical specimens and plasmid standards by paper capture, denaturation, and detection (CDD) and plate CDD for mutations K103N and Y181C. Panels A and C show mutant (MUT) detection, and Panels B and D show wild-type (WT) detection. Sample optical density (OD) minus negative control OD (left y axis) for each specimen is shown in white/gray by rank along the x axis, from the lowest MUT OD, followed by the plasmid standards (0%, 5%, 50% MUT) performed in duplicate. Spot intensity minus background intensity (right y axis) for each specimen is shown in blue and green bars followed by the plasmid standards (0%, 5%, 50% MUT) performed in triplicate. Scanned images of the paper CDD detection strip are shown below each specimen’s signal data.

**Fig 5:**
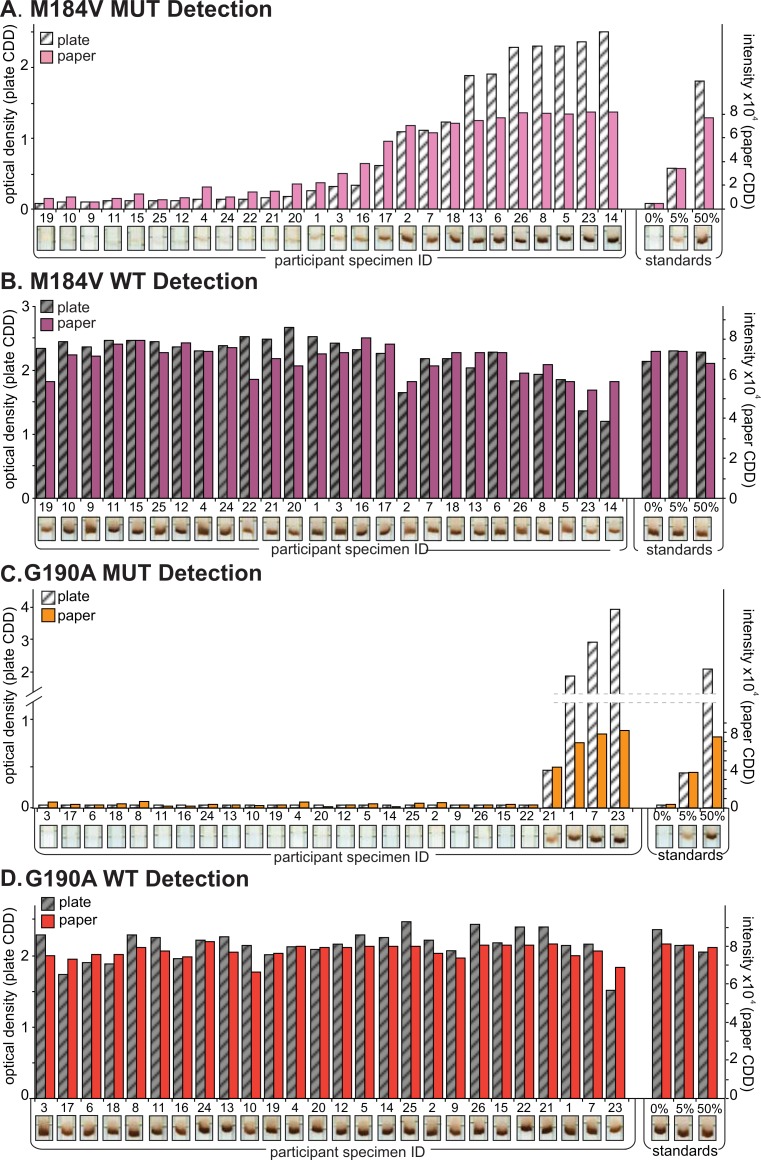
Analysis of clinical specimens and plasmid standards by paper capture, denaturation, and detection (CDD) and plate CDD for mutations M184V and G190A. Panels A and C show mutant (MUT) detection, and Panels B and D show wild-type (WT) detection. Sample optical density (OD) minus negative control OD (left y axis) for each specimen is shown in white/gray by rank along the x axis, from the lowest MUT OD, followed by the plasmid standards (0%, 5%, 50% MUT) performed in duplicate. Spot intensity minus background intensity (right y axis) for each specimen is shown in pink and orange bars followed by the plasmid standards (0%, 5%, 50% MUT) performed in triplicate. Scanned images of the paper CDD detection strip are shown below each specimen’s signal data.

**Fig 6:**
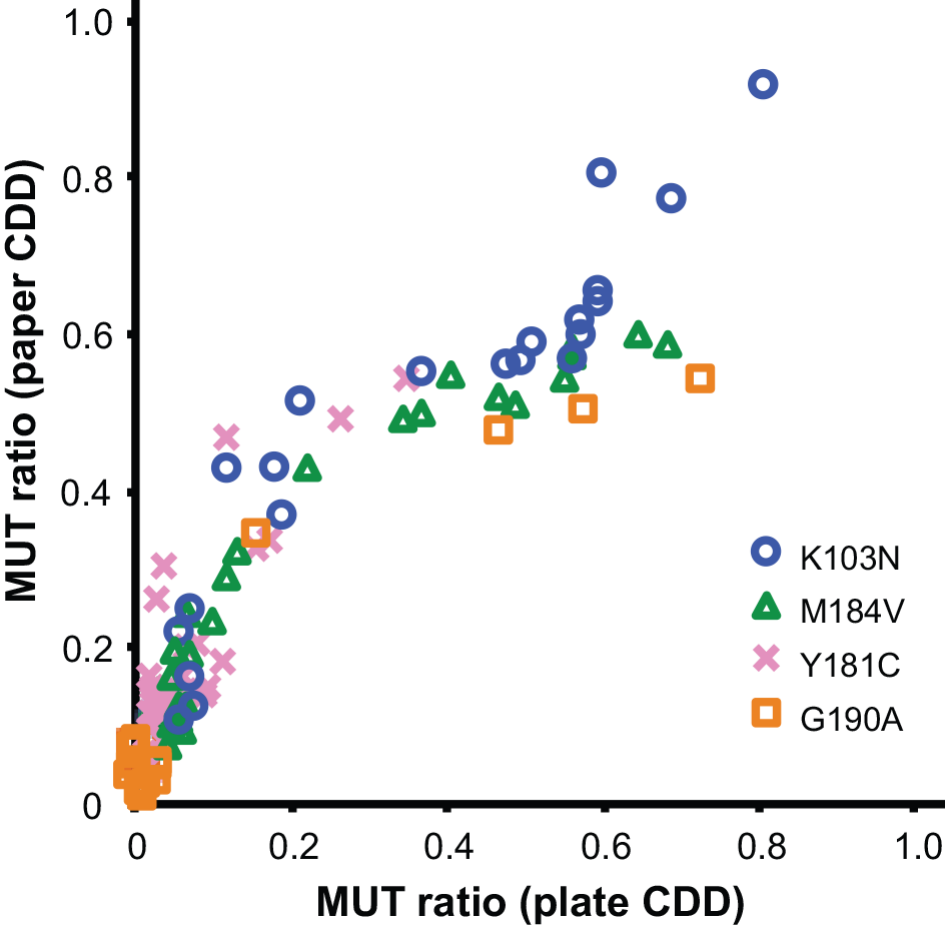
Correlation plot of MUT Ratios determined by paper capture, denaturation, and detection (CDD) versus plate CDD. MUT and WT signals obtained by paper and plate CDD format OLA at codons K103N, Y181C, M184V and G190A from 26 clinical specimens were used to calculate MUT Ratios. The overall shape reflects the signal saturation of the paper CDD at high MUT concentration, as seen in Figs 3–5. Results correlate strongly across clinical specimens and plasmid standards’ data (see S4 Fig for fitted correlation).

Clinical specimens (N = 26) from Kenyan’s infected with HIV-1 subtypes A, C, D, AE and G, and plasmid standards at 0, 5 and 50% MUT were tested for drug resistance mutations at four HIV codons (K103N, Y181C, M184V and G190A, N total = 104) by both paper CDD and plate CDD OLA (Figs 4 and 5). At low % MUT, the MUT assay showed a linear correlation between both CDD formats for specimens, while signal saturated in the paper CDD at high % MUT (S4A–S4D Fig). The WT assay showed strong responses for most specimens and plasmid standards in both formats (S4E–S4H Fig). For all codons combined, the MUT Ratios for paper CDD and plate CDD correlate strongly across participants’ data (Fig 6). The polynomial fit of data is shown in S4I–S4K Fig.

## Discussion

In this work, the detection procedure (i.e., CDD) of the OLA-based DR HIV test was simplified to enhance adoption in low-resource settings. The primary modification was conversion of CDD from a plate format to a paper strip format. Exploiting rapid capture and labeling in paper reduced reaction time for all binding steps (5 minutes each). Instead of two-stage enzymatic labeling for WT and MUT detection used in the plate OLA, we simultaneously detected WT and MUT using the same enzyme system (i.e., POD) in parallel strips (with the same timing of steps). Additionally, use of a precipitating substrate (i.e., DAB) created visible spots. This eliminates the need for a spectrophotometer to determine the threshold signal for a positive reaction and to estimate the proportion of mutant in subjects’ specimens.

The plate CDD uses streptavidin-coated well plates to capture ligation probes and a denaturing buffer to remove non-ligated probes from template DNA. In the paper OLA, streptavidin was immobilized onto nitrocellulose strips via spotting and drying. We found that a more stringent denaturing buffer (10X compared to plate CDD) was needed to achieve low background (S2 Fig). The 10X denaturing buffer did not strip streptavidin from the nitrocellulose (S3A Fig, One-Way ANOVA, α = 0.05), and had little impact on its ability to capture a biotinylated target (S3C Fig, statistically significant but small effect).

The paper strip format reduced the time needed to perform the assay. Typical plate assays require long incubation steps for target capture and labeling, due in large part to slow diffusion of probes and other reagents to the plate capture surface. In contrast, delivery by flow in paper provides intimate contact of reagents with the detection surface, as targets and labels diffuse rapidly across the small pores (mean of 2.8μm [25]). As a result, capture and labeling of ligated products could be completed within 5 minutes each in the paper CDD, compared to long incubations (1-hour and 30-minute incubations, respectively) in the plate CDD.

In the plate CDD, the POD system produces detectable signal much faster (5 minutes) than the AP system (15–45 minutes), and the two detection steps for MUT and WT are performed sequentially in a single well. To improve speed of detection in the paper format, a single enzyme system (POD) was used to label MUT and WT probes in parallel strips run simultaneously using the same procedure. TMB is the most common substrate for POD-based plate assays, but it requires addition of H_2_SO_4_ to stop the reaction prior to reading [26], and the soluble TMB product [27] would be washed away in the paper CDD. DAB is extensively used to measure peroxidase activity in tissues [28]; it has only one oxidation state (does not require post-treatment to fix the color) and forms visible brown precipitate at the detection spots on the paper strip. The replacement of AP by POD for MUT detection and the use of DAB substrate likely caused the differences between the plate and paper signal saturation seen with all specimens and standards. The oligonucleotide probe concentrations had been optimized in the plate CDD for the slower AP-yellow substrate system, and probe concentrations were not adjusted in the paper CDD. However, a linear correlation can likely be achieved in the paper CDD by adjusting the concentrations of oligonucleotide probes. If detection of low MUT HIV levels is needed to predict treatment failure, tuning CDD for strong low-level response may be desirable.

The relatively large SD values on the standard control samples were greater for codons 103 and 181 compared to 184 and 190. The small number of standard control samples (triplicate) was sensitive to variation, and the variability in these experiments was likely due to manual spotting of the paper strips instead of controlled strip preparation. Controlled strip preparation (e.g., lateral flow striper) is expected to reduce the variability. Overall, the paper CDD showed analytical sensitivity comparable to the plate CDD for a plasmid mixture series (Y181C, Fig 3 & S4I Fig), and results for clinical specimens showed strong concordance across four relevant DR codons (Fig 6 & S4J Fig).

While we did not attempt to evaluate % MUT quantification here, our results suggest a quantitative correlation with plate CDD, which correlates strongly with proportions of mutant in the HIV population as measured by Roche’s 454 pyrosequencing [22]. In addition, drug resistance surveillance and treatment monitoring requires a test that targets all DR mutations prevalent in the patient population. Here, we tested three common nevirapine- and one lamivudine-resistance mutations currently prevalent in Kenya [10]. With the availability of new drug choices for first-line ART, additional DR mutations such as K65R will likely be needed to more comprehensively assess transmitted and selected HIV DR. The clinical utility of the test will require evaluation based on available treatment options. Notably, the paper CDD is expected to translate well to new DR mutations since the capture and detection chemistries are not target-dependent. OLA can be performed on amplicon derived from either plasma viral RNA or cellular DNA (PBMC, whole blood, dried blood spots) [17–20]. In this study, we used PBMC DNA as the HIV DR target as DNA-based assays offer several advantages over RNA, such as cost savings from reverse transcriptase enzyme and easier handling and processing (no need for RNAse-free reagents and plastic/glass ware). In addition, for pre-ART testing of DR, DNA is adequate and can detect archived resistance mutations no longer detectable in plasma [21,29]. However, testing for DR in plasma RNA may be more sensitive in patients with early rebound viremia. The existing plate CDD measures WT and MUT in sequential reactions within a single well. In the paper CDD, we separated the WT and MUT detection into two strips run in parallel. This reduced the protocol time and complexity, but a procedural control may be needed to ensure the validity of each strip.

The modifications developed here simplified the CDD steps and reduced the total time. Both plate and paper CDDs share the upstream processes (DNA extraction, amplification, and ligation), which take 420min. Paper CDD reduced the total OLA time from 420min plus 150min/ 1 DR codon to 420 min plus 45 min/ 1 DR codon. The costs for the complete OLA processes with plate and paper CDDs are comparable (~ $5/codon, S5 Table). Note that the costs reported represent batching of 36 and 9 samples for plate and paper CDDs, respectively. More potential simplifications on paper CDD can be done. For example, the CDD reagents could be stored dry without refrigeration and supplied in wells, as reagent pads integrated into strips [30] similar to conventional lateral flow tests, or printed directly onto the strip [31]. Long-term storage of some CDD reagents have been demonstrated: DAB substrate [32], POD-conjugated antibodies [33,34]. While the paper CDD presented here requires manually moving the paper strips between wells every 5 minutes, there is potential to automate the timed steps required in the CDD protocol. Paper-microfluidic tools such as dissolvable sugar time delays [35], shunt time delay valves [36,37], and capillary-based paper networks [38,39] could enable automation of the protocol presented here. Increasing simplicity of the paper CDD through dry reagents and automation of the protocol is the subject of current work.

Finally, here we addressed only the CDD portion of the assay, but specimen preparation, amplification, and ligation still require laboratory procedure and tools, including a thermal cycler. However, the CDD procedure has been the most difficult portion of the OLA procedure to transfer to new laboratories, and simplifications to CDD al one should improve access to OLA. Future work also includes simplification and improvement of other steps in OLA, for example, nucleic acid extraction and concentration to improve assay sensitivity for low HIV copy samples as well as isothermal amplification and ligation to avoid the need for a thermal cycler.

## Conclusions

A simple low-cost test for HIV DR could improve efficacy of global ART programs, allow better control of the HIV pandemic, reduce the selection and transmission of drug resistance, and prolong the usefulness of limited drug regimens in resource-constrained regions. The OLA allows identification of HIV drug resistance at a lower cost and higher sensitivity than Sanger sequencing, but the complexity of the protocol has been an obstacle for implementation. We have reduced the time and complexity of the most complex step of the OLA–the detection procedure. The paper CDD offered more than a three-fold reduction in procedure time (2.5 hours to 45 minutes) and a reduction in procedure complexity, while yielding performance comparable to the current plate CDD for detection of DR HIV in clinical specimens. While the paper CDD still relies on procedures and instruments for amplification and ligation, the simplification of the detection protocol presented here could allow easier transfer of the OLA to middle-tier to low-tier laboratories, where resources are more limited.

## Supporting Information

S1 Fig Correlation plots of paper capture, denaturation, and detection (CDD) and plate CDD for analysis of plasmid standard mixtures for mutation Y181C. (A) Optical density (OD) of MUT from plate CDD versus intensity of MUT from paper CDD (B) OD of WT detection from plate CDD versus intensity of WT detection from paper CDD (C) MUT ratio from plate CDD: OD MUT/(OD WT + OD MUT) versus MUT ratio from paper CDD: intensity MUT/(intensity MUT + intensity WT).
(EPS)

S2 Fig Denaturing of non-ligated probes to remove background. (A) Schematic of efficient oligonucleotide denaturing. DNA target is completely dissociated from probes and false signal is not detected. (B) Schematic of inefficient oligonucleotide denaturing. Non-ligated probes bound to target template DNA can be detected, contributing to false signal. (C) Comparison of denaturing methods. 1X and 10X concentration of denaturing buffer were tested for their ability to denature non-ligated probes from target template DNA compared to a 1X PBST control. To generate complete non-ligated probes, a 0% mutant (MUT) plasmid standard of Y181C was mixed with probes and subjected to 10 cycles of ligation in the absence of ligase enzyme. 1X denaturing buffer was not effective at reducing background (11% reduction), but the 10x denaturing buffer was effective (94% reduction).
(EPS)

S3 Fig On-paper oligonucleotide capture by immobilized streptavidin. **(**A) Streptavidin presence on membrane after 1X PBST or 10X denaturing buffer wash. The presence of streptavidin immobilized on nitrocellulose was visualized by Ponceau S solutions (10 minutes in Ponceau S solution and 5 minutes in distilled water). The wet strips were scanned and analyzed. The plot represents signal intensity of stained streptavidin in each case (mean ±SE, n = 4). There was no significant different between means (one-way ANOVA, p = 0.07) (B) Determination of linearity of biotin-conjugated-POD (biotin-POD) concentrations captured by on-paper streptavidin. Biotin-POD was used as a model analyte to quantify capture performance. 25μL of 10-fold serial dilutions of biotin-POD/ PBST (6.25pg to 62.5μg) was used as a model analyte (n = 4). The strips were dipped in a timed sequence (5 minutes each) in polystyrene wells containing the following reagents: (1) 25μL of biotin–POD, (2) 25μL of PBST wash, (3) 40μL of DAB substrate, and (4) 25μL of PBST wash. Wet strips were scanned and analyzed. The plot represents signal intensity (mean ± SE, n = 4). The linear range was estimated as 0.1–10μg of biotin-POD, and 625ng of biotin-POD was chosen for the experiment in C (such that an effect, if present, would be observed by a change in signal intensity). (C) Effect of denaturing buffer on the ability of immobilized streptavidin to capture biotin-POD. The strips were dipped in a timed sequence (5 minutes each) in a series of polystyrene wells containing the following reagents: (1) 25μL of 1X PBST, 1X denaturing buffer, or 10X denaturing buffer, (2) 25μL of neutralizing buffer (twice), (3) 25μL of 625ng of biotin-POD (within the responsive range from panel B), (4) 25μL of PBST, (5) 40μL of DAB substrate, and (6) 25μL of PBST. On-paper streptavidin after 10X denaturing buffer yielded significantly lower signal intensity than the ones treated with PBST or 1X denaturing buffer (One-way ANOVA, Post-hoc comparison (Tukey Kramer test, α = 0.05).
(EPS)

S4 Fig Correlation Plots. (A)-(D) MUT signal between paper CDD and plate CDD for the data in Figs 4A & 3C and 5A & 4C, respectively. (E)-(H) WT signal between paper CDD and plate CDD for the data in Figs 4B & 4D and 5B & 5D, respectively. (I) Correlation plot of Y181C Mutant (MUT) Ratios for 26 subjects’ HIV DNA compared to the plasmid mixtures (0%–100% Y181C MUT) and specimen. (J) Correlation plot of MUT Ratios for all four HIV-drug-resistance codons determined by paper capture, denaturation, and detection (CDD) versus plate CDD as presented in Fig 6. In both plots, a polynomial fit was used to report R^2^ values for the correlations. (K) Explanation of the non-linear curve in B. For samples containing low % mutant (zone 1), the MUT signals dominated the MUT Ratios because the WT signals were saturated in both plate and paper CDDs. Paper CDD is more sensitive at low % mutant than the plate CDD, and this results in a slope >1. The MUT signal of paper CDD reached the saturation earlier than the plate CDD, which causes a plateau-like region (zone 2). As WT signals were less saturated in samples with high % mutant, the slope increases (zone 3).
(EPS)

S1 Table Oligonucleotide probe sequences and concentration in ligation. (EPS)

S2 Table Quantitative data of signal ratios of plasmid standards and clinical specimens evaluated by plate capture, denaturation, and detection (CDD). (EPS)

S3 TableCalculation of signal intensity in paper CDD and MATLAB code for image analysis.Red, green, and blue pixels of the image were first projected onto an L-2 vector [0.684 0.1705 0.792], which was developed by linear discriminate analysis to best separate the color of DAB signal from its background on nitrocellulose, and signals were reported as intensity (range: 0–3^0.5^ x 2^16^A.U., Eq 1). The reported signal intensity is calculated using Eq 2.
$${\left[\text{intensity}\right]}_{\left(55\;\text{x}\;40\right)\;\text{x}\;1}=\;{\left[\text{ROI pixel intensity}\right]}_{\left(55\;\text{x}\;40\right)\;\text{x}\;3}\times {\left[\text{L}-2\;\text{unit vector}\right]}_{3\;\text{x}\;1}$$(Eq 1)
$\text{signal intensity}\;=\;95^{\text{th}}\;\text{percentile of}\;{\left[\text{intensity}\right]}_{\text{background}}-95^{\text{th}}\;\text{percentile of}\;{\left[\text{intensity}\right]}_{\text{signal}}$(Eq 2)(EPS)

S4 Table Quantitative data of signal ratios of plasmid standards and clinical specimens evaluated by paper capture, denaturation, and detection (CDD). (EPS)

S5 Table Comparison of time, cost and instrument requirements for plate and paper CDDs as well as the other steps in OLA. (EPS)
